# Combined orbits and clocks from IGS second reprocessing

**DOI:** 10.1007/s00190-018-1149-8

**Published:** 2018-05-18

**Authors:** Jake Griffiths

**Affiliations:** Naval Center for Space Technology, U.S. Naval Research Laboratory, Washington, DC USA

**Keywords:** IGS, GPS, Reprocessing, Orbits, Clocks, Combination results

## Abstract

The Analysis Centers (ACs) of the International GNSS Service (IGS) have reprocessed a large global network of GPS tracking data from 1994.0 until 2014.0 or later. Each AC product time series was extended uniformly till early 2015 using their weekly operational IGS contributions so that the complete combined product set covers GPS weeks 730 through 1831. Three ACs also included GLONASS data from as early as 2002 but that was insufficient to permit combined GLONASS products. The reprocessed terrestrial frame combination procedures and results have been reported already, and those were incorporated into the ITRF2014 multi-technique global frame released in 2016. This paper describes the orbit and clock submissions and their multi-AC combinations and assessments. These were released to users in early 2017 in time for the adoption of IGS14 for generating the operational IGS products. While the reprocessing goal was to enable homogeneous modeling, consistent with the current operational procedures, to be applied retrospectively to the full history of observation data in order to achieve a more suitable reference for geophysical studies, that objective has only been partially achieved. Ongoing AC analysis changes and a lack of full participation limit the consistency and precision of the finished IG2 products. Quantitative internal measures indicate that the reprocessed orbits are somewhat less precise than current operational orbits or even the later orbits from the first IGS reprocessing campaign. That is even more apparent for the clocks where a lack of robust AC participation means that it was only possible to form combined 5-min clocks but not the 30-s satellite clocks published operationally. Therefore, retrospective precise point positioning solutions by users are not recommended using the orbits and clocks. Nevertheless, the orbits do support long-term stable user solutions when used with network processing with either double differencing or explicit clock estimation. Among the main benefits of the reprocessing effort is a more consistent long product set to analyze for sources of systematic error and accuracy. Work to do that is underway but the reprocessing experience already points to a number of ways future IGS performance and reprocessing campaigns can be improved.

## Introduction

In early 2015, the International GNSS Service (IGS; Dow et al. 2009) Analysis Centers (ACs) completed a second reanalysis of Global Navigation Satellite System (GNSS) data collected for a global network of tracking stations. This second reprocessing, or repro2, updates the set of definitive IGS combination data products—station positions; satellite orbits and clocks; and Earth orientation parameters (EOPs)—using the latest analysis models and methodologies. The data used for repro2 spanned $\sim 21$ years, starting January 2, 1994, and continuing through February 14, 2015, or GPS Weeks 730 through 1831. Participating analysis groups used data for the US Global Positioning System (GPS) and also the Russian GLONASS system in the case of three ACs. This paper focuses on results from the repro2 orbit and clock combinations, resulting in reprocessed IGS combined (IG2) orbit and clock products for GPS only. Please refer to Rebischung et al. (2016) for a description of the tracking network used and the associated reprocessed station positions and EOPs.

### Historical context

To date, two IGS reprocessing campaigns have occurred outside but in parallel with the IGS operational product streams (ultra-rapid, rapid, final) and were generally undertaken with the goal to advance Earth science research through updates to the International Terrestrial Reference Frame (ITRF). Repro2 provided the IGS input (Rebischung et al. 2016) to ITRF2014 (Altamimi et al. 2016). It followed the successful first reprocessing campaign, or repro1, which provided the IGS input for ITRF2008 (Altamimi et al. 2011). The resulting reprocessed combination orbit and clock products plus the follow-on Finals operational products aim to disseminate the latest realization of the ITRF without a loss of fidelity. Therefore, the IGS reprocessing campaigns nominally aim to homogenize the full history of IGS combination data products in an internally consistent way, with ACs adopting the latest analysis models and methodologies available at the time of the campaign. In that approach, a posteriori quality assessments of the resulting reprocessed combination products can serve to inform future reprocessing campaigns and help to advance the state-of-the-art while providing quantitative measures of product accuracy. This is the ideal scenario; the realities are sometimes quite different. Often the ACs disagree on a common set of analysis models to be used or simply fail to apply timely changes, and that can lead to reduced internal precision and complicate interpretations of the combination results. This will become evident as repro2 orbit and clock combination results are discussed in the subsequent sections.

**Fig. 1:**
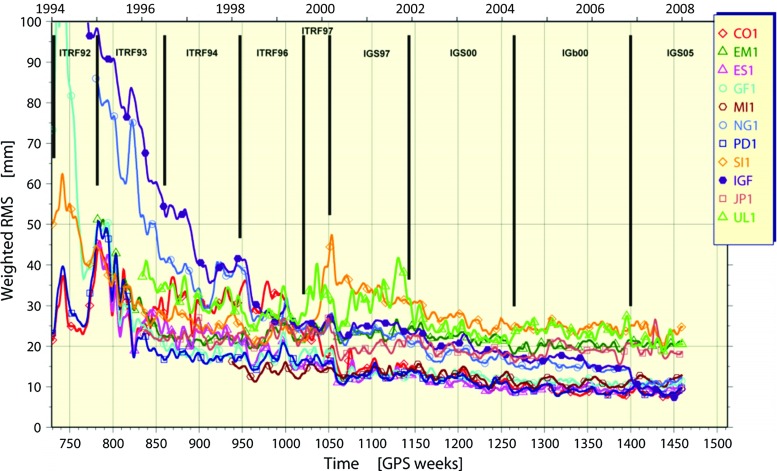
Time series of AC weighted RMS (WRMS) statistics from the repro1 orbit combination (Gendt et al. 2010). PD1 is for the Potsdam/Dresden group, which contributed as an extra solution. SI1 is for the solution contributed by Scripps Institution of Oceanography, an operational IGS AC. The WRMS statistics are computed using the post-fit residuals of the combination for each contributed solution. The curve for IGF represents the WRMS difference for the operational IGS Finals orbits compared to IG1 orbits from repro1. The steady and dramatic decline in the IGF curve represents the accumulated effect of errors being removed in the operational products through the series of historical analysis changes. The IGF orbits were included for comparison only; they did not contribute to IG1. Black vertical lines along the top of the chart mark indicate when new terrestrial frames were adopted for the operational products

The idea that IGS reprocessing campaigns aim to homogenize the full history of IGS combination data products presumes that somehow heterogeneities appear in the established set of products at some point. It is not hard to see how those heterogeneities can get introduced. The evolution of the original IGS Finals operational data products (IGF) prior to repro1 is a good example. After the operational product streams were established in the early days of the IGS, the ACs naturally began adopting new analysis models and methods incrementally for their operational products. Mostly these newly adopted models led to improved AC products and therefore improved the IGS combination products going forward. But as analysis improvements compounded, the oldest products were less useful because they were less precise and inaccurate. Eventually, use of the earlier products in Earth science research began to limit studies of long-term geophysical processes. This was exacerbated in particular by periodic adoption of updated global reference frames and changes in calibration tables for ground and satellite antennas. It became clear that reprocessing the full history of data offered the potential to remove past heterogeneities, improve the IGS contribution to the ITRF and potentially advance Earth science research. For repro1, a set of common standard models and methods was established, and the operational ACs focused for several years on implementing them into their software. A full summary of those repro1 analysis standards is available online (http://acc.igs.org/reprocess.html), but the main changes were due to:

- a switch to absolute calibrations (Gendt, IGSMAIL-5272; Schmid et al. 2007) for receiver antennas and GLONASS and GPS satellite transmitter antennas;
- adoption of the IGS05/igs05.atx framework (Ferland 2006, IGSMAIL-5447; Ferland and Piraszewski 2009), which is aligned to ITRF2005 (Altamimi et al. 2007); and
- general implementation of the International Earth Rotation and Reference Systems Service (IERS) 2003 Conventions (McCarthy et al. 2003), including updated models for tropospheric propagation delay and for station displacements due to ocean tidal loading with whole-Earth center-of-mass corrections applied to SP3 orbits.

**Fig. 2:**
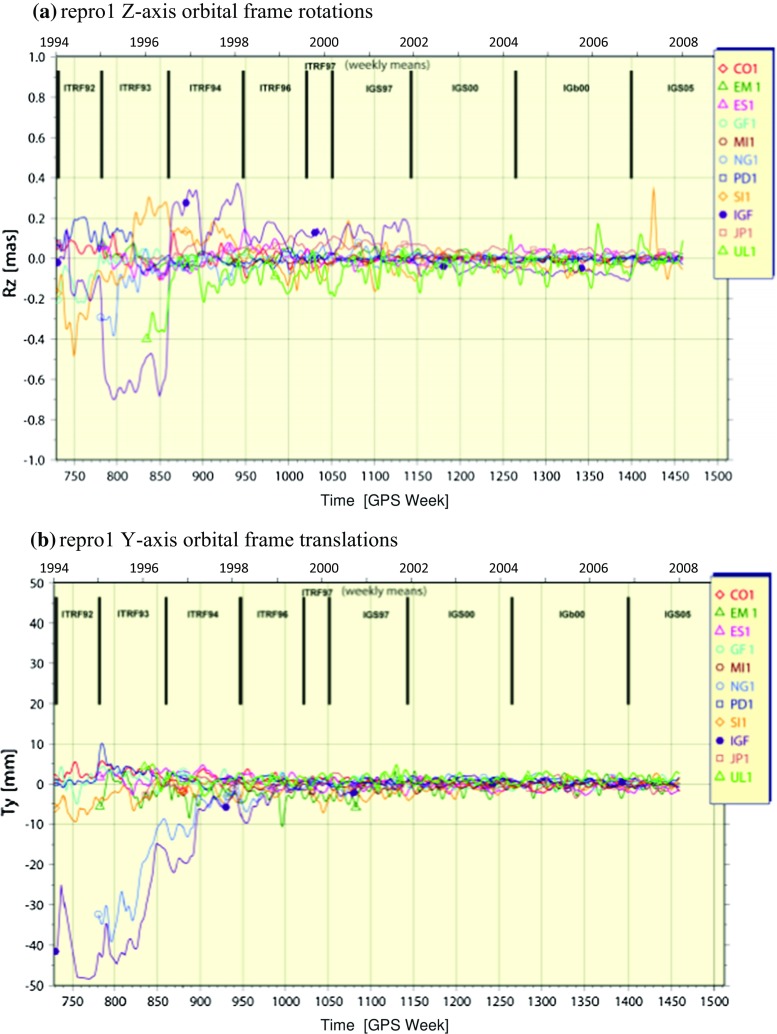
In addition to WRMS statistics for post-fit residuals in Fig. 1, the orbit combination estimates AC orbital frame transformation parameters with respect to the combined orbit. **a** Time series of Helmert orbit frame rotations about the Z-axis for AC repro1 contributions and IGF, and **b** time series of Helmert origin translations in the Y-direction for the same AC solutions (Griffiths et al. 2009). The two plots in (**a**, **b**) illustrate historical frame errors removed with the adoption of IGS05 and reprocessing the full history of GPS data for the IG1 data products

Figure 1 shows WRMS time series statistics (smoothed) for each contributing AC in the repro1 orbit combinations. The trace of the IGF curve shows the evolution of the WRMS difference compared to IG1 and demonstrates historical improvements in the internal precision of the historical IGS operational products. The step decrease at GPS Week 1400 (November 05, 2006) is due to the switch to absolute antenna calibrations when the IGS05 reference frame was adopted for the operational product streams (Gendt, IGSMAIL-5438; Schmid et al. 2009). Other discrete analysis changes happened at other times and are less obvious in the WRMS statistics. For instance, the many IGS reference frames adopted over the years did not obviously impact the IGF WRMS statistics despite the fact that adopting a new frame is a discrete event. However, dramatic impacts from several of the frame changes are clearly seen in the Helmert rotation and translation parameters of some ACs (e.g., Fig. 2a,b) estimated in the repro1 orbit combination (Gendt et al. 2010).

### Known remaining errors in IGS products

As Figs. 1 and 2 illustrate, repro1 marked an overall dramatic improvement in the orbit precision and long-term frame stability compared to the original IGS Finals operational products. As will be discussed in the following sections, the incremental improvements made for repro2 following repro1 are less dramatic because the overall errors in the IG1 products are already much smaller than they were in the original operational products. However, while the overall accuracy and precision of the IGS definitive products reached a remarkable level with repro1, significant deficiencies continue to affect the products at the few centimeters level and smaller (see Ray 2016 for a recent review). The largest of the errors include a combination of: unattributed subseasonal errors (Ray et al. 2013); effects of background power-law noise in station coordinates on station velocities (Zhang et al. 1997; Santamaría-Gómez et al. 2011); effects of positional discontinuities on ground station velocities and frame stability (Williams 2003; Griffiths and Ray 2016); effects on EOPs and station time series residuals due to terrestrial frame misalignments to a long-term reference (Ray et al. 2017); subdaily EOP alias and draconitic errors in the satellite orbits (Griffiths and Ray 2009, 2013) and those plus other harmonics in time series of ground station positions (Ray et al. 2008; Rebischung et al. 2016), which have been attributed to a combination of local near-field multipath, mismodeling of solar radiation pressure (SRP) and possibly other orbit-related errors; and various annual signals including unmodeled station displacements due to surface pressure loading, temporal changes in the low-degree geopotential coefficients and thermal expansion and flexure of ground antenna structures, among others. Some of these errors, like the background power-law noise in station coordinates, may be intrinsic to GPS. Others, like draconitic and tidal alias errors, can possibly be mitigated with continued efforts to update analysis models. Still other errors arise from inattentive operational and managerial diligence (e.g., excessive positional offsets caused by equipment changes, poor siting of tracking antennas and missing metadata needed to compute thermal variations). In any case, most of these significant errors are expected to persist in the repro2 combination products and probably beyond.

### IGS repro2 analysis models

A full list of analysis standards used for repro2 is summarized online (http://acc.igs.org/reprocess2.html), but the main changes from repro1 are:

- a switch from weekly to daily terrestrial frame integrations to facilitate the study of station displacements at higher temporal resolution (Griffiths 2012, IGSMAIL-6613; Griffiths and Choi 2013; Rebischung et al. 2013);
- inclusion of GLONASS data by some (three) but not all ACs;
- the implementation of the IGb08/igs08.atx reference frame and calibration framework (Rebischung et al. 2012, IGSMAIL-6354, IGSMAIL-6663);
- general implementation of the IERS 2010 Conventions (Petit et al. 2010), and of particular note the addition of the ocean pole tide displacement and the change to the cubic plus linear conventional model for the mean pole motion;
- higher-order (at least 2nd order) ionospheric and updated tropospheric models for propagation delays;
- implementation of new attitude models for eclipsing satellites (Kouba 2009; Dilssner 2010; Dilssner et al. 2011) by some but not all ACs; and
- modeling of Earth radiation pressure (Rodriguez-Solano et al. 2011a, b) including also the thrust acting on the spacecraft caused by signal transmission along the satellite antenna boresight (Rodriguez-Solano et al. 2012; acc.igs.org/orbits/thrust-power.txt).

As with repro1, ACs were asked not to apply model corrections for the load displacements caused by large-scale non-tidal atmosphere, ocean and hydrological surface fluid motions. The primary seasonal components of the load effect on station positions and EOPs, as well as all other annual and semiannual signals, were removed empirically by explicit fitting in the subsequent long-term stacking process used by Altamimi et al. (2016) to form ITRF2014.

While the above repro2 standards were generally implemented, some AC software changes departed from the recommended standards (see Table 2 for explanation of AC abbreviations). The main known departures are:

- COD
  - assumes nominal attitude during eclipses for GPS and GLONASS (applies to operational products only since broadcast clocks provided in CF2 SP3 files)
  - ocean pole tide not applied (displacements pre-applied for SINEX combination)
- EMR
  - assumes nominal attitude during eclipses for GPS Block IIF satellites; other GPS satellites use yaw-steering model
- ESA
  - assumes nominal attitude during eclipses for GPS
  - no modeling of Earth albedo and antenna thrust
  - ocean pole tide not applied (displacements pre-applied for SINEX combination)
  - 2nd-order ionospheric correction not applied
- GFZ
  - site displacements due to atmospheric S1/S2 tidal loading applied without applying associated center-of-mass offset corrections to SP3 file
- GRG
  - site displacements and SP3 center-of-mass corrections due to atmospheric S1/S2 tidal loading applied
- JPL
  - 30-hour data spans and orbital arcs
- MIT
  - ocean pole tide not applied (displacements pre-applied for SINEX combination)
  - SRP parameters constrained between days over 9 d window
  - applied non-tidal atmospheric pressure loading in processing; effects removed from SINEX
  - no modeling of orbit perturbations due to ocean tidal geopotential variations

**Table 1:** AC contributions for IGS repro2, along with IGS products (IG1/F) used for comparison only. The italic rows in columns 3 and 4 contain the product acronym and time spans of the AC repro2 solution. The bold rows highlight the segments of operational AC products used to extend repro2 solutions through GPS Week 1831

AC	Institution	Product acronym	Time spans (yyyy-mm-dd)
COD	Center for Orbit Determination in Europe	*cf2*	*1994-01-02 to 2013-12-28*
		**cof**	**2013-12-29 to 2015-02-14**
EMR	Natural Resources Canada	*em2*	*1994-10-02 to 2014-03-29*
		**emr**	**2014-03-30 to 2015-02-14**
ESA	European Space Agency	*es2*	*1995-01-01 to 2014-04-19*
		**esa**	**2014-04-20 to 2015-02-14**
GFZ	GeoForschungsZentrum Potsdam	*gf2*	*1994-01-02 to 2015-01-17*
		**gfz**	**2015-01-18 to 2015-02-14**
GRG	Groupe de Recherche en Géodésie Spatiale	*gr2*	*1998-01-01 to 2014-12-31*
		**grg**	**2015-01-01 to 2015-02-14**
JPL	Jet Propulsion Laboratory	*jp2*	*1994-01-02 to 2014-10-25*
		**jpl**	**2014-10-26 to 2015-02-14**
MIT	Massachusetts Institute of Technology	*mi2*	*1994-01-02 to 2014-08-02*
		**mit**	**2014-08-03 to 2015-02-14**
GTZ	GeoForschungsZentrum Potsdam (TIGA)	*gt2*	*1994-01-02 to 2012-12-29*
ULR	Université de la Rochelle (TIGA)	*ul2*	*1995-01-01 to 2013-12-31*
IG1	IGS repro1 products	*ig1*	*1994-01-02 to 2007-12-29*
IGF	IGS operational Finals products	*igs*	*2007-12-30 to 2015-02-14*

**Fig. 3:**
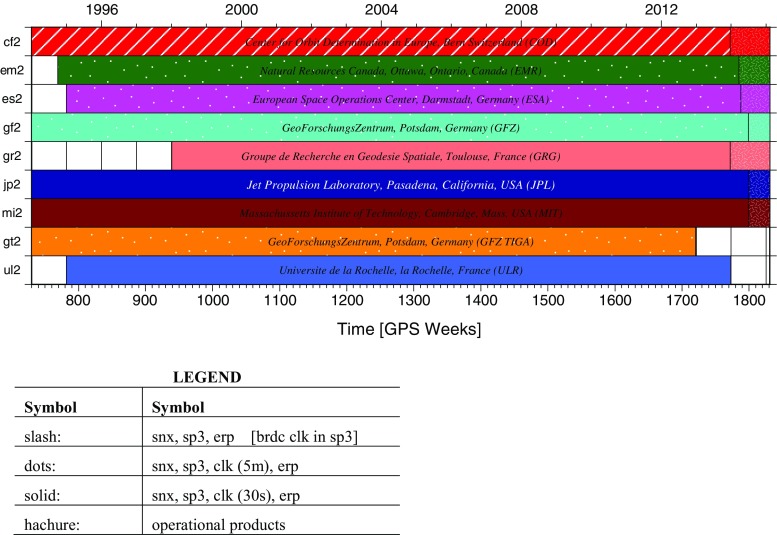
Graphical representation of the repro2 orbit and clock inputs described in Table 1

Some of the departures listed are small and have limited implications for the combination products. Others, like the application of day-boundary constraints on SRP parameters by MIT, have unknown effects. Still others have potentially dramatic impacts, depending on weighting of the offending AC product in the combination. For instance, radial orbit errors caused by neglecting the effects of ocean tidal geopotential variations can reach 20 centimeters on average over the long term for a geodetic satellite in low earth orbit (Petit et al. 2010). For GPS, the impact is $<0.1\,\hbox {mm}$ on average over the long term, but subdaily perturbations can exceed 42 centimeters 3D RSS. So, the effects are of course dampened for GPS satellites due to their altitude, but are still significant for high-accuracy applications. Also, the lack of consistent yaw modeling among clock ACs has a large impact on the satellite clock combination. As a practical matter, mixing input clock solutions derived with differing yaw models complicates usage of the IGS combination clock product because the user is unable to match their own selected model with that of the IGS clocks.

At least one AC made changes to their operational products since their repro2 submissions. ESA made several orbit modeling changes, iterating on the most suitable box-wing model for GPS satellites (Springer 2017). A change was made to their operational products at GPS Week 1892, dropping altogether the box-wing model for GPS Block IIF. This put the ESA orbits into strong alignment with JPL until about GPS Week 1938 (http://acc.igs.org/igsacc_final.html). Consequently, and also because the National Geodetic Survey (NGS) and Scripps Institute of Oceanography (SIO) ACs did not contribute to the repro2 effort, the consistency between the IG2 products and the follow-on operational Finals is not optimal.

### Repro2 AC contributions and usage

Nine analysis groups submitted solutions for repro2 (Table 1): seven IGS Finals operational ACs and two centers from the IGS tide gauge benchmark monitoring working group (TIGA; Schöne et al. 2009), which primarily serve to densify the tracking network with GNSS stations that are co-located with tide gauges, tying the tide gauge measurements directly to ITRF2014. However, one operational AC and both TIGA centers were ultimately excluded from the orbit and clock combinations for reasons described below. As Table 1 and Fig. 3 show, each contributed solution spans a different segment of time. They start as early as January 2, 1994, and extend to at least the end of 2013. Generally, those end dates correspond to when an AC completed their repro2 software changes in their IGS Finals operational products. For the TIGA groups, the time spans were determined by their internal group-specific requirements. AC operational products were used to extend AC solutions uniformly through GPS Week 1831. The approach outlined in Table 1 is consistent with Table 1 of Rebischung et al. (2016) for the IG2 station products.

**Table 2:** AC contributions explicitly excluded from the repro2 orbit (SP3) and clock (CLK) combinations

AC	Product	Time spans (yyyy-mm-dd)	Remarks
GRG	SP3	All	Excluded in SINEX; large orbit and clock biases
	CLK	All	
MIT	SP3	1994-01-02 to 2003-12-20	0.5 ppb scale biases; tests show small impact after 2003-12-14
	CLK	1994-12-17 to 2015-02-14	$>250$ ps biases and many jumps, used for a few weeks when not enough ACs
GTZ	SP3	All	Redundancy with GFZ
	CLK	All	
ULR	SP3	All	Included in SINEX except for geocenter; large orbit and clock biases
	CLK	All

**Table 3:** Official IG2 orbit and clock products, where GPS Week, *wwww*, ranges from 0730*to*1831, and day-of-week ranging, *d*, from 0*to*6. There is not an internally realized IG2 timescale; the IG2 clock products are linearly aligned to GPST via the broadcast clocks each day. Daily erps in weekly accumulated files along with long-term accumulated erp files are listed too

File	Description
ig2[wwww][d].sp3	Daily orbit SP3c files
ig2[wwww][d].clk	Daily station and *5-min* clk RINEX files (there are no IG2 *30-sec* satellite clks)
ig2[wwww][d].cls	Daily clk combination summary files
ig2[wwww]7.erp	Daily ERPs in weekly concatenated files
ig2[wwww]7.sum	Weekly orbit, clock, ERP combination summary
ig215p01.erp	Accumulated ERP file from orbit and clock combo
ig215p02.erp	Definitive accumulated ERP file from SINEX combo

AC repro2 orbits and clocks were used to form the combination products in a way that closely matches what was done in forming the IGS combined SINEX files (Rebischung et al. 2016). This was done to maintain consistency between the orbital and terrestrial reference frames expressed by the joint set of IG2 products (Table 2). One exception is the use of GFZ orbits and clocks instead of GTZ. This was done for purely conventional reasons because the GFZ products contribute to the operational product stream and the GFZ orbits and clocks are nominally identical to those of GTZ. The tracking network used for GTZ was simply expanded to include TIGA stations in the GTZ submission. This decision has no measurable impact on the combination results. The other exception to the IG2 SINEX selections is in the handling of MIT contributions to the orbit and clock combinations. Biases exceeding 0.50 ppb in their orbit scale and many clock jumps and biases exceeding 500 ps should preclude their use entirely, but excluding their orbits over the full repro2 span introduces a 5-mm WRMS disparity in IG2 orbits compared to IG1. So MIT orbits were included after GPS Week 1249 in order to minimize discrepancies with the IG1/F products in the earlier years^1^. The MIT clocks were also problematic due to large biases and frequent jumps. They were largely excluded, though they were used to fill-in gaps in the combination for the earliest years (GPS Weeks 730-779 and 1111-1115) because there are too few good AC clock solutions to form a robust and precise IG2 CLK product. Moreover, no 30-sec satellite clock combinations were formed due to an insufficient number (i.e., $<3$) of useable (e.g., detectable jumps and removable biases) AC solutions.

**Fig. 4:**
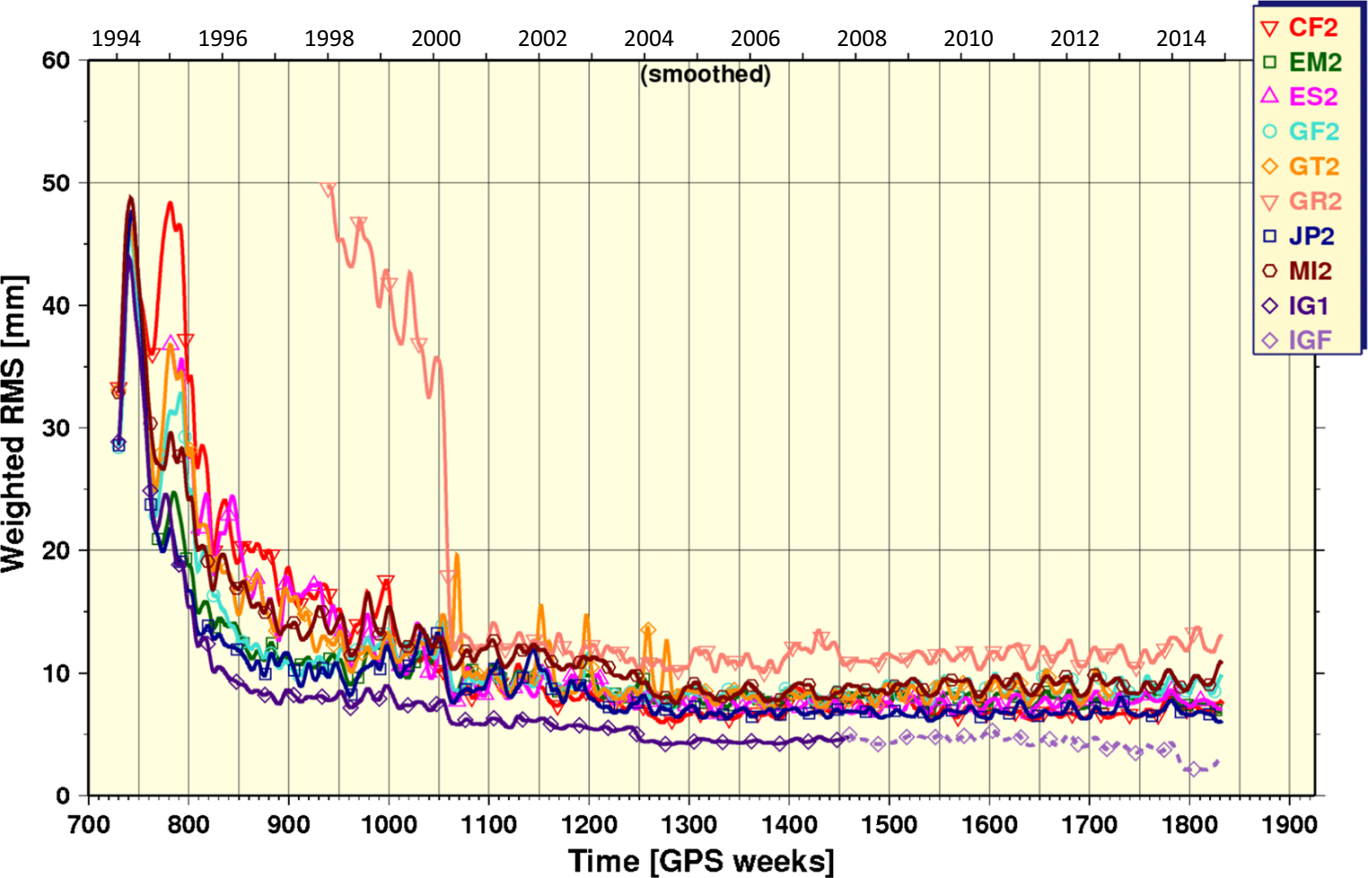
WRMS statistics for repro2 AC post-fit residuals from the orbit combination. The IG1 and IGF orbits do not contribute to the combined IG2 orbits; they were included for comparison only. While UL2 orbits were also included for comparison only, the UL2 residuals exceeded 110 mm and do not fit in the scale of this plot

## IG2 orbit and clock combinations

The combination models used for generating the IG2 orbits and clocks are identical to those used by the Analysis Center Coordinator (ACC; acc.igs.org) in generating the IGS operational Final products. The underlying orbit combination model has been the same since the early days of the IGS (Beutler et al. 1995; Kouba et al. 1995; Kouba and Mireault 1996, 1997), though a change to the combination pre-processing steps was introduced in early 2000 in order to co-align the IGS orbital and terrestrial reference frames (Kouba et al. 1998; Ferland et al. 2000, IGSMAIL-2740; Ferland et al. 2000; Springer 2000, IGSMAIL-2750). The change meant applying small Helmert rotations, determined from the IGS SINEX combination, to the AC Final orbits prior to combining them into the IGS Final orbits. During the switch to daily SINEX integrations in 2012, however, a bug in the combination software was discovered. The sets of SINEX TRF rotational offsets used for co-aligning the AC orbits were being applied with incorrect signs (Griffiths 2012). Correcting the software bug starting with GPS Week 1702 (August 19, 2012) eliminated spurious rotations of the IGS Final orbital frame, which reached $100\,{\upmu }$as about the Y-axis. The combination model and strategy used for the clocks are the one introduced by Kouba and Springer (2001).

As with the IG1 reprocessed products, the full suite of repro2 orbit and clock combination products (see Table 3) are available the IGS Global Data Center at NASA (i.e., ftp://cddis.gsfc.nasa.gov/gps/products/*wwww*/repro2 or  ftp://cddis.gsfc.nasa.gov/gps/products/repro2). The *ig2wwww7.erp* and *ig215p01.erp* files are from the ACC orbit and clock combination system. They are derived for the purpose of monitoring the official ERP products generated in the SINEX combination (Ferland et al. 2000; Rebischung et al. 2016). The main differences between the ACC and SINEX ERPs are the use of *a posteriori* AC orbit residual statistics as a priori weights to the AC ERPs and the absence of station covariances when they are combined in the ACC system. The definitive EOP products are provided by the Reference Frame Combination Center at the Institut National de l’Information Géographique et Forestière (IGN) in France and are the ones to be used with the orbits.

### Orbit combination statistics

In addition to the resulting combined ephemerides, the orbit combination generates post-fit residuals for each AC after removing seven Helmert transformation parameters (rotation, translation, scale) for each daily input orbit relative to the daily combined orbit. The time series of daily WRMS of the input orbit residuals computed from the repro2 combination results are shown in Fig. 4 (smoothed). The WRMS in Fig. 4 are akin to those in Fig. 1, but for the repro2 inputs. With exception of UL2 and GR2, the WRMS agreement of the input solutions and IG2 trends approximately exponentially over the repro2 time span. The WRMS agreement between IG1/F and IG2 improves from $\sim 28\,\hbox {mm}$ in the earliest years converging to $\sim 5\,\hbox {mm}$ at GPS Week 1250. Their mutual agreement improves further to about $\sim 2.5\,\hbox {mm}$ over a time span of about 27 weeks, from GPS Week 1773 (December 29, 2013) until about GPS Week 1800 (July 6, 2014). Recall that this is the period when COD, EMR and ESA operational products were introduced to the repro2 combinations (Table 1). These are three of the more highly weighted inputs in the operational Finals combinations (http://acc.igs.org/igsacc_final.html). The remaining 2.5-mm WRMS difference between IG2 and IG1/F after GPS Week 1800 is attributed mostly to the absence of a repro2 solution from the NGS AC (the missing SIO AC solution having little impact). This result suggests that the absence of an NGS repro2 solution introduces a background difference in IG2 compared to IG1/F at the $\sim 2.5\,\hbox {mm}$ WRMS level.

**Fig. 5:**
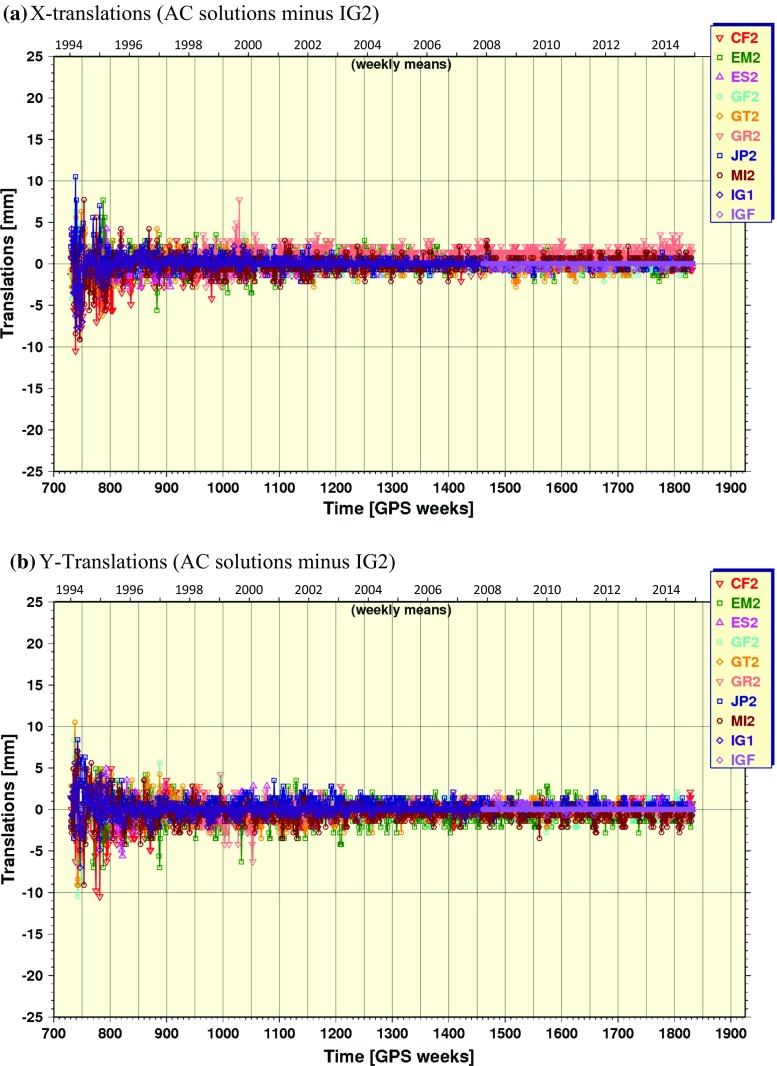
Time series of orbital frame origin translations estimated in the repro2 orbit combination in the **a** X-direction, **b** Y-direction and **c** Z-direction of the IG2 orbital frame

### Orbital frame

#### Translations

Helmert orbital frame translations estimated in the repro2 orbit combinations are plotted in Fig. 5 for each geocentric coordinate. All ACs have nearly annual motions in the Z-translation with various phases, but approximately similar amplitudes, except for GRG whose annual motions sometimes reach 20 mm for unexplained reasons. Meanwhile, the equatorial translations are nearly featureless for all ACs.


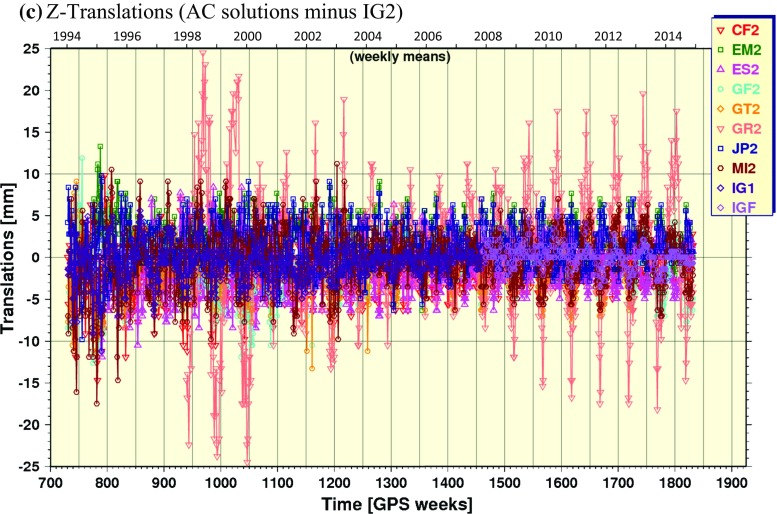


#### Rotations

**Fig. 6:**
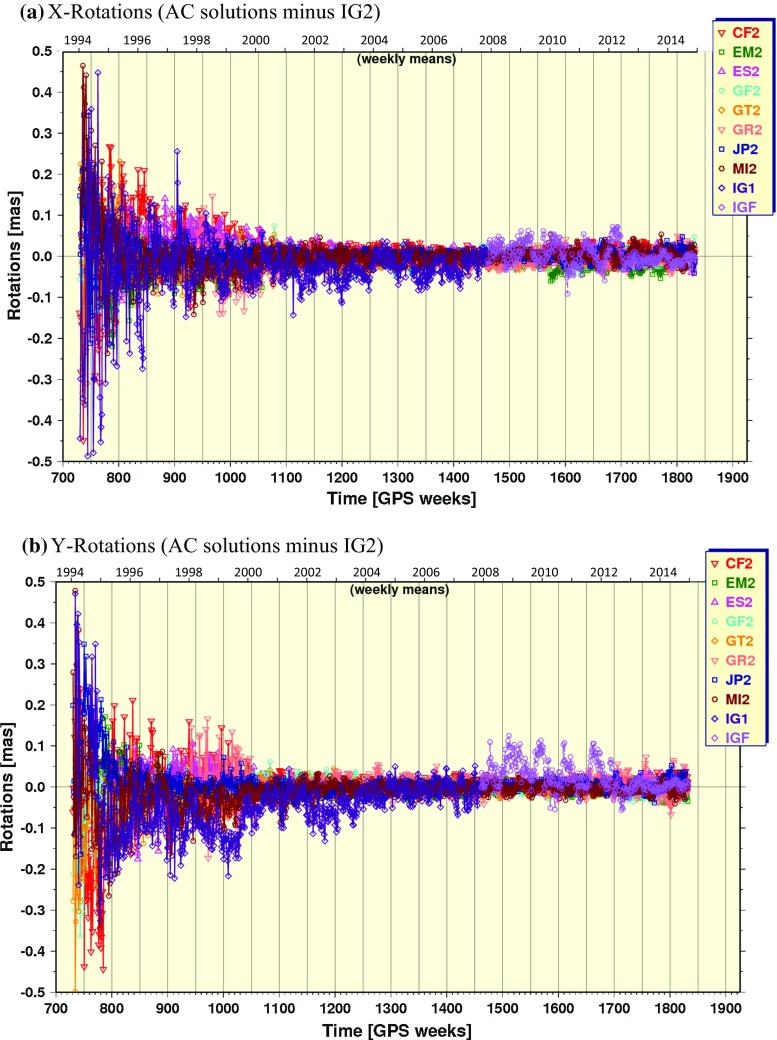
Same as in Fig. 5, but for rotations about the geocentric **a** X-axis, **b** Y-axis and **c** Z-axis


Helmert rotations about each geocentric axis coordinate from the repro2 orbit combinations are plotted in Fig. 6. Large variations sometimes occur about all three axes for most ACs prior to GPS Week 1150 (January 20, 2002). The most striking result, however, is the large (up to $100\,{\upmu }$as) Rx and Ry offsets for IG1/F prior to GPS Week 1702, which was caused by the ACC frame alignment software bug described by Griffiths (2012) and mentioned earlier.

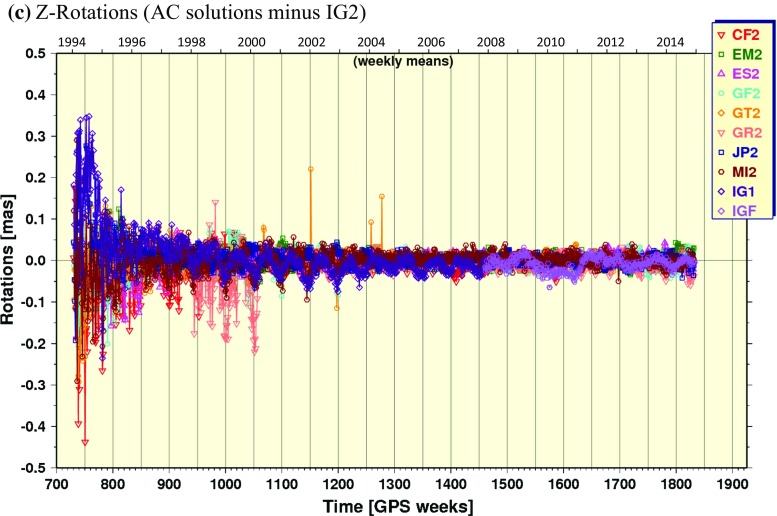


#### Scale

**Fig. 7:**
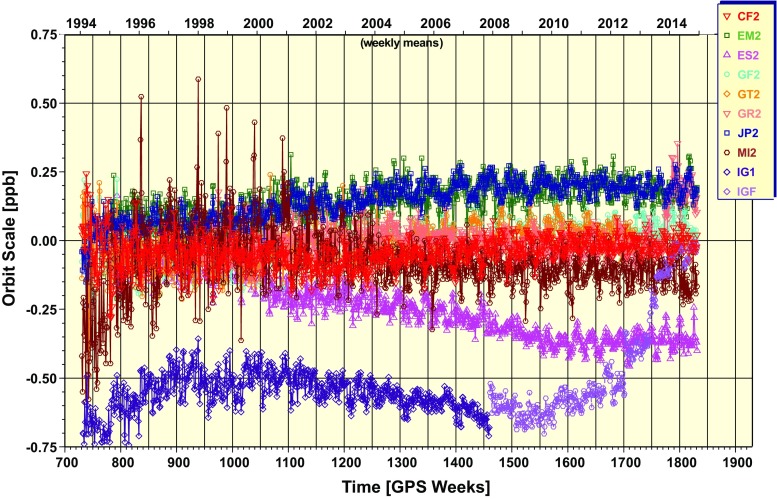
Time series of Helmert scale offsets estimated in the repro2 orbit combination

Helmert scale offsets estimated in the series of repro2 orbit combinations are plotted in Fig. 7. MIT orbits have large spurious offsets prior to GPS Week 1250 (December 21, 2003), but the MIT products were excluded during this period so they do not impact the IG2 orbit scale. The EMR and JPL orbital scales track each other closely because they both use GIPSY-OASIS for their data analysis. ESA and IG1/F vary similarly for GPS Weeks 1050–1550, but then afterward drift independently. The average scale offset of $\sim 0.55$ ppb (1.46 cm at GPS) between IG2 and IG1/F before Week $\sim 1700$ is smaller than the $\sim \,0.72$ ppb (Ray 2012) offset expected from adopting Earth albedo and antenna thrust models in the repro2 analyses. The missing 0.17 ppb part of the IG2 scale change is attributed primarily to the fact that ESA did not adopt these two orbit modeling changes for repro2.

The IG1/F scale converges toward IG2 starting at GPS Weeks 1702 (August 19, 2012), which, as mentioned above, coincides with the switch to daily products and the software fix for applying AC SINEX rotations to the AC orbits. The faster convergence of IG2 and IG1/F after GPS Week 1740 (August 21, 2011) is likely due to operational ACs adopting the repro2 Earth albedo and antenna thrust models in their contributions to the operational IGS Finals products. With the exception of ESA, these model changes were completed by about GPS Week 1773 (December 29, 2013).

### Clock combination statistics

**Fig. 8:**
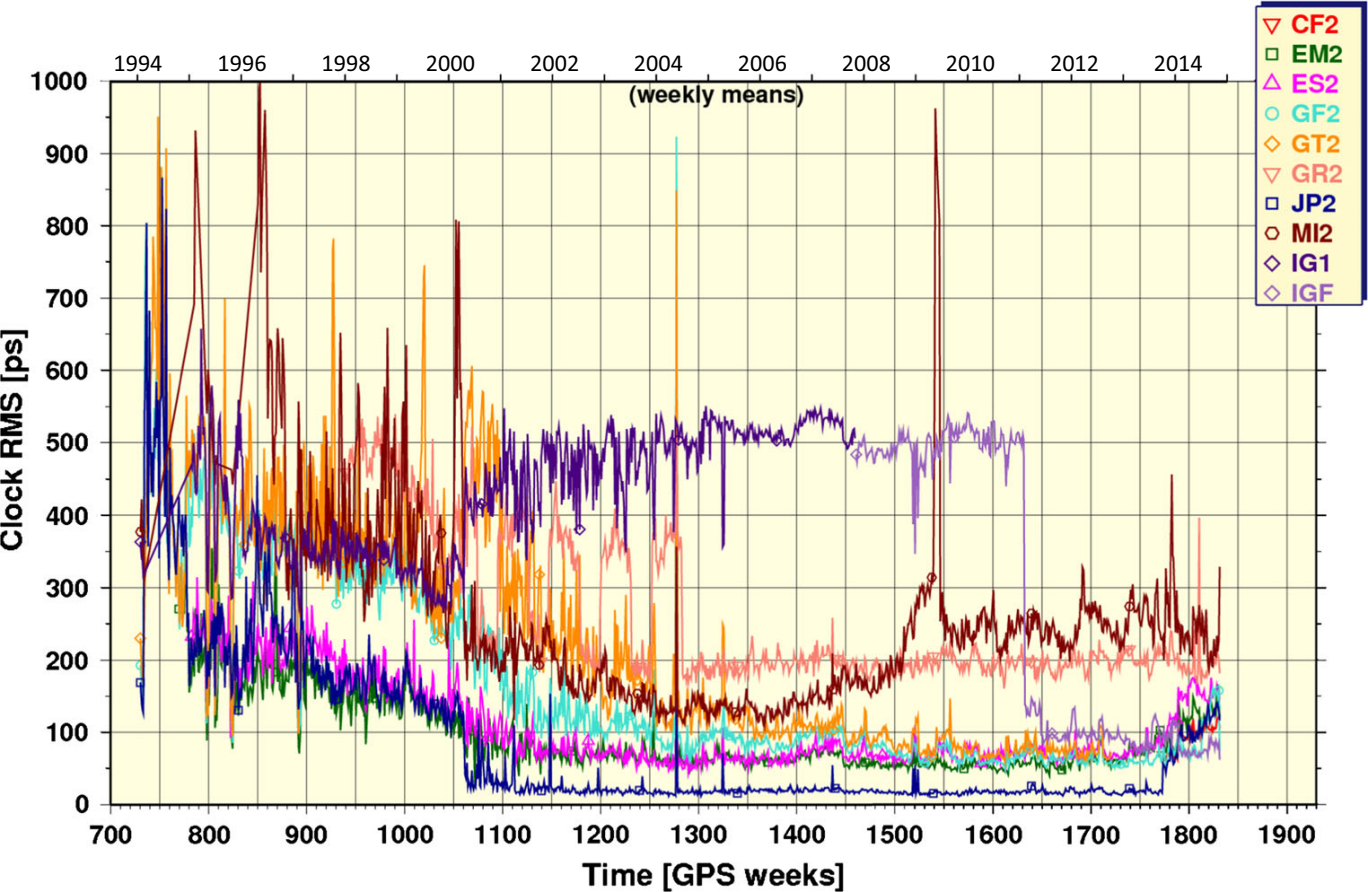
Ensemble clock RMS values for each solution in the combination

**Fig. 9:**
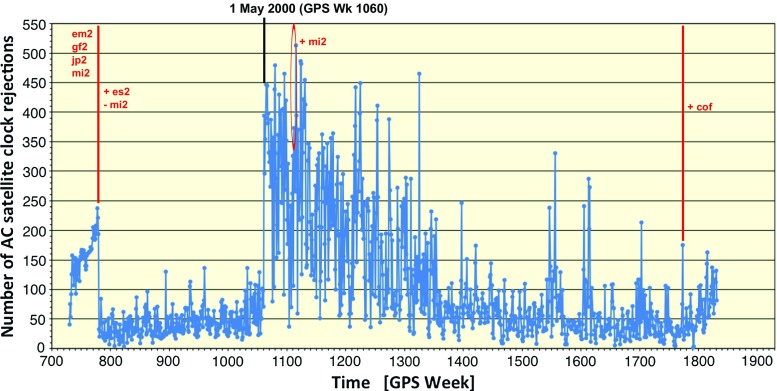
Number of AC GPS satellite clock rejections per week in the combination. The rejections are determined per epoch of the clock sampling, so $\sim 500$ rejections compared to clock estimates for 32 satellites sampled at 5-minute intervals, for instance. The same rejection criteria were used for the entire combination period. The ACs contributing to the clock combinations are listed, with the earliest years beginning with EM2, GF2, JP2 and MI2. MI2 was given zero weight when ES2 was added at GPS Week 780, except for when MIT was used to fill-in gaps between GPS Week 1111 and 1115. The CODE operational clocks (COF) were added at GPS Week 1772. The number of rejections is especially large for the period between GPS Weeks 1060 and 1335 (or May 1, 2000, and August 14, 2005). May 1, 2000, has some significance because that is the date when the US Air Force turned off Selective Availability (SA). It is unlikely that turning off SA is responsible for the spike in AC clock rejections. Rather, better inter-AC clock agreement in the pre-SA-off period is relative to some background noise level. In the pre-SA-off era, when there is a tremendously large noise background, it is likely that the clock combo algorithm simply cannot detect when an AC clock value is bad or not, except when they are egregious. On the other hand, when SA gets turned off, the background satellite clock noise drops by a very large amount and the algorithm is able to better detect discrepant AC values

Figure 8 shows weekly averages for the RMS statistics of individual repro2 AC clock solutions with respect to the combined 5-min clock product. The RMS statistics include separate biases for each satellite and station clock, which are computed and removed before generating the final IG2 clocks, when possible. The step in the combination process that computes the biases requires three reliable (few jumps, distinguishable biases) input clock products. When the number of reliable clock products falls below three, the ability to reliably determine the clock biases fails and a large number of AC clock rejections can occur (see for example Fig. 9). When the step for determining AC biases fails, the combination becomes unstable and the combined clock product gets contaminated with AC biases, which appear as spikes in the AC RMS curves like those near GPS Week 1523 in the JP2 series. The step decrease in the RMS curve for IGF at GPS Week 1631 (April 17, 2011) corresponds to when the IGS switched from the IGS05 to IGS08 framework (including station coordinates and antenna calibrations) in the IGS Finals operational product stream. The step increase in the RMS curve for JP2 at GPS Week 1773 (December 29, 2013) corresponds to when the CODE operational clocks (and orbits) were incorporated into the repro2 combination. Recall from Fig. 3 that CODE did not contribute repro2 clock estimates.

It was mentioned earlier that the clock combination requires at least three good input AC solutions to form a robust product. Another complicating factor negatively impacting the combined clocks is the fact that ESA assumes nominal attitude through satellite eclipse season. Other ACs adopted a yaw-steering model, but not necessarily the same one. During these periods the resulting clock inconsistencies cause automatic rejections to increase in the combination, which can sometimes become unstable and unreliable if insufficient numbers of usable clock ACs remain.

## Quality assessment of the IG2 orbits and clocks

Determining the quality of the repro2 products provides insights into their utility for accessing the IGS reference frame for high-accuracy positioning, navigation and timing applications, as well as indicating the progress of AC modeling efforts. While there is no method to *validate* IGS orbits, several approaches have been developed for detecting systematic errors. Analysis of positional discontinuities at the midpoint epoch between successive daily SP3 files (Griffiths 2009a, b; Griffiths and Ray 2009) has been extremely valuable for detecting limiting errors in the IERS Conventions model for subdaily EOP variations (Griffiths and Ray 2013), among other things. A refined version of that 2013 analysis is currently underway for the IG2 orbits and is the subject of a manuscript in preparation. Satellite laser ranging (SLR) has also been used to great effect (e.g., Urschl et al. 2005; Ziebart et al. 2007; Sośnica et al. 2015, 2017). An anonymous reviewer of this paper used IG2 orbits to reprocess International Laser Ranging Service (ILRS; ilrs.org) SLR data for the two GPS satellites (SNV35 and SVN36) equipped with a laser retroreflector array. They apparently found that the RMS difference between the optical and microwave ranges is reduced by 1.5 cm compared to those when using IG1/IGF orbits. That result is consistent with the main IG2–IG1/IGF orbit scale offset discussed earlier (Fig. 7), which was an expected result given the repro2 orbit model changes for Earth albedo and satellite antenna thrusting. The reviewer also found that the standard deviation of SLR residuals decreased by 1–2 mm, which may be due to improved rotational stability of the orbital frame after correcting the combination software bug discussed earlier. One must be cautious, however, not to overstate SLR’s role in validating orbit accuracy. *Validation* is a very high standard. With ranging precisions approaching 1 mm, SLR is highly sensitive to variations in the orbit radial direction—the most accurately modeled orbit component due to Kepler’s 3rd law—but SLR is relatively insensitive to along-track and cross-track errors^2^. Moreover, the SLR technique continues to suffer other limitations affecting its accuracy, as illustrated by long-standing issues with range biases (e.g., Appleby et al. 2016). This is compounded by the fact that SLR to GPS is limited to two old satellites with relatively infrequent observations that ended in 2013.347 (SVN35) and 2015.835 (SVN36). All of this ignores inter-technique modeling errors inherent in the IERS Conventions (Petit et al. 2010), which for GPS can exceed 2.5 cm in the along-track and cross-track directions (Griffiths and Ray 2013). For all of these reasons, the next two subsections are focused on results from a long-arc orbit analysis, following Griffiths and Ray (2009), and a precise point positioning (PPP) analysis to determine the utility of IG2 orbit and clock products for long-term reprocessing by IGS users.

**Table 4:** Averages and standard deviations of the daily median statistics of the 1D residuals from the long-arc analysis

	Average (± SD) of daily long-arc medians (mm)	
Product	Full repro2 time span	Since GPS Wk 1336 (2005-08-14)
IG1/F	24.4 ($\pm \,11.6$)	19.4 ($\pm \,3.7$)
IG2	26.5 ($\pm \,8.9$)	22.8 ($\pm \,3.0$)

### Long-arc analysis

Estimates of orbit precision were derived from a long-arc analysis of each weekly set of IG2 and IG1/F orbits, where seven days of combined SP3c files are fit for 15 parameters of the extended CODE orbit model (ECOM; Springer et al. 1998)—a six-parameter orbit state vector (position and velocity) and the nine terms of an empirical harmonic SRP forcing model expressed in a satellite body-fixed frame. Then RMS, WRMS and median statistics are computed for the orbit residuals on a daily basis. These statistics are reported in the companion *.sum files noted in Table 3 of Section 2. This procedure is identical to that used for the operational Finals summaries.

Table 4 shows the ensemble averages and standard deviations for the daily median statistics from the long-arc analysis for IG1/F and IG2 orbits. These ensemble statistics are computed over the full repro2 time span (GPS Weeks 0730 thru 1831, or 1994.003 thru 2015.121) and are taken to represent the internal long-term precision of the IG2 and IG1/F orbits. The question is whether the precision of IG2 is significantly different from that of IG1/F. A z-test comparing the average median from IG2 and IG1/F indicates that the precision of the two solution series is indeed significantly different at the 99.9% confidence interval, and that IG2 is less precise than IG1/F.

The root of the square differences for the values in Table 4 over the full time span is computed to approximate the magnitude in the loss of precision for IG2:

$$\begin{aligned} \hbox {RDS}_{\mathrm{medi}} = 7.31\,\hbox {mm} =\sqrt{{\left| {\hbox {Medi}_{\mathrm{IG2}}^{2}-\hbox {Medi}_{\mathrm{IG1/F}}^{2}} \right| }\Big /{2}} \end{aligned}$$

As mentioned earlier, the orbit combination results (Fig. 4) indicate that about half of the WRMS discrepancy between IG2 and IG1/F can be attributed to not having a repro2 contribution from the NGS AC. It then follows that half of the 7.31-mm loss in precision for IG2 is attributable to the absence of an NGS repro2 solution. That is, not having a repro2 solution from NGS introduces a significant discrepancy with respect to IG1/F and reduces the overall precision of the IG2 orbits. In that case, the remaining half of the 7.31 mm $\hbox {RDS}_{\mathrm{medi}}$ are likely due to other errors in other highly weighted AC orbits (i.e., EMR, ESA, JPL) that do not appear to exist in their operational solutions. For instance, errors committed in ES2 solutions associated with using a box-wing a priori solar radiation pressure model (Springer et al. 2014) that was later found to be erroneous (Springer 2017).

**Table 5:** Same as Table 4, but for the ACs contributing to the IG2 combinations with nonzero a priori weight along with a brief description of the SRP modeling approach used. The subtle AC model and methodology differences complicate detailed interpretation. But CF2 and EM2 offer the clearest result, where no clear bias favoring the ECOM model is seen

Product	Average (± SD) of daily long-arc medians (mm)	Remarks about SRP modeling
CF2	31.9 ($\pm \,16.3$)	No a priori; ECOM 6+5
EM2	30.9 ($\pm \,7.0$)	Empirical a priori, small stochastic adj.; 24-hr data arc
ES2	27.6 ($\pm \,10.0$)	Box-wing a priori plus ECOM 6+5
GF2	31.5 ($\pm \,15.7$)	No a priori; ECOM 6+5
JP2	29.5 ($\pm \,8.1$)	Empirical a priori, small stochastic adj.; 30-hr data arc
MI2	27.5 ($\pm \,7.7$)	ECOM 6+9, with 9d constraints

**Table 6:** Average and standard deviations for post-fit residuals after Helmert transformation comparison of a PPP network of coordinates to the IG2 daily terrestrial frames, which are expressed in IGb08. Statistics are given over the full repro2 time span and for a more recent subset in order to gauge their utility over the last decade of repro2. The smaller biases and scatter in the station position residuals indicate higher precision in the latter span of the products, but the IG2 products are still not as precise as IG1/F

Product	East (mm)	North (mm)	Up (mm)
Full IG2 time span			
IG1/F	4.3 ($\pm \,0.96$)	2.3 ($\pm \,0.82$)	6.4 ($\pm \, 1.43$)
IG2	4.5 ($\pm \,1.39$)	2.7 ($\pm \, 1.43$)	6.9 ($\pm \,2.04$)
Since GPS Wk 1336 (2005-08-14)			
IG1/F	3.9 ($\pm \,0.70$)	2.0 ($\pm \, 0.56$)	5.8 ($\pm \,1.07$)
IG2	4.0 ($\pm \,0.80$)	2.1 ($\pm \,0.74$)	6.1 ($\pm \, 1.25$)

Of course, there is a question as to whether ECOM is appropriate for the IG2 long-arc analysis given inter-AC differences for SRP modeling. That is, perhaps ECOM (Beutler et al. 1994) is simply more consistent with the IG1/F orbits because a so-called reduced-parameter version (i.e., 6-parameter state vector and five terms of the full nine term truncated Fourier series) of ECOM is the most common among the ACs, and so the differences in the long-arc statistics largely reflect model inconsistency. We have no way to answer this question directly. However, the large number of degrees of freedom in the full ECOM should be adequate to represent the IG2 orbits for this analysis. The AC-specific long-arc results in Table 5, where no bias in favor of ACs using ECOM is seen, seem to support this assertion. Therefore, the author believes it is the IG2 orbits that are less precise even for the second half of the repro2 time span. This loss in precision impacts PPP, but probably not results where double differencing is used because the errors should be largely common mode and minimized or eliminated by the differencing technique.

### PPP analysis

A PPP analysis was performed to assess the utility of the IG2 orbit and clock products as a means for accessing the IGS reference frame in a long-term PPP reprocessing by generating coordinate sets consistent with IGS08 and transforming them to IGS14 via the IERS transformation parameter values (Altamimi et al. 2016). The overall analysis approach follows the one used operationally by the IGS ACC. The Bernese v5.2 software (Dach et al. 2015) was employed to estimate daily positional coordinates for 163 core IGS14 stations (Rebischung et al. 2016, IGSMAIL-7399) separately using IG1/F and IG2 orbit and clock products. IERS 2010 Conventions are generally implemented, including GMF/GPT2 (Böhm et al. 2006; Lagler et al. 2013) troposphere modeling with daily tropospheric delay gradients following Chen and Herring (1997). Plus,

- RINEX files for each IGS14 core station were downloaded from NASA/CDDIS (cddis.gsfc.nasa.gov);
- daily SP3 satellite files were fit using IG2 official EOPs and extended CODE (6+9) orbit model in order to provide satellite positions at the sampling interval of the data files;
- IG2 clocks and station data were preprocessed to detect and eliminate clock jumps and cycle slips;
- code and phase observational data were reduced with iterative post-fit cleaning; outliers are phase residuals exceeding 25 mm and code residuals exceeding 2.5 m;
- floating-point phase ambiguity parameters were estimated; and
- daily coordinate sets were compared to IG2 combined daily SINEX solutions, which were aligned to IGb08 by Rebischung et al. (2016), resulting in daily sets of PPP-derived terrestrial frame parameters and station coordinate residuals.

Compared to IG1/F, the IG2 orbits and clocks deliver long-term global coordinate sets that are noisier and maybe slightly more biased (Table 6). The added noise in the long-term PPP results is caused by a high number of station data rejections in the PPP processing prior to GPS Week 1336. This period corresponds to when there were large numbers of satellite clock rejections in the clock combination (Fig. 9), implying that the AC input clocks were of poor quality that degrades the PPP results.

**Fig. 10:**
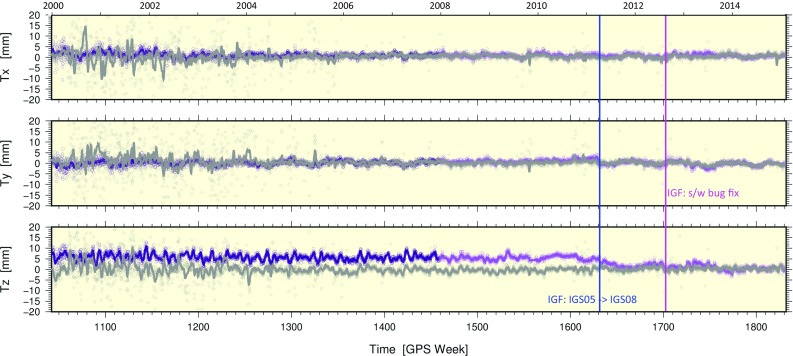
PPP-derived frame translations for IG2-based (gray) and IG1/F-based (purple) solutions compared to IG2 SINEX frames that are aligned to IGS08 (Rebischung et al. 2016). The solid curves are from a 7d boxcar average. The large scatter appearing as noise in the earlier weeks of the gray series reflects instability of the PPP-based IG2 frame caused by large numbers of station rejections (due to satellite clock issues). The blue vertical line marks the switch from the IGS05 frame to IGS08 in the IGF operational stream. The magenta vertical line marks the timing of the s/w bug fix in IGF

**Fig. 11:**
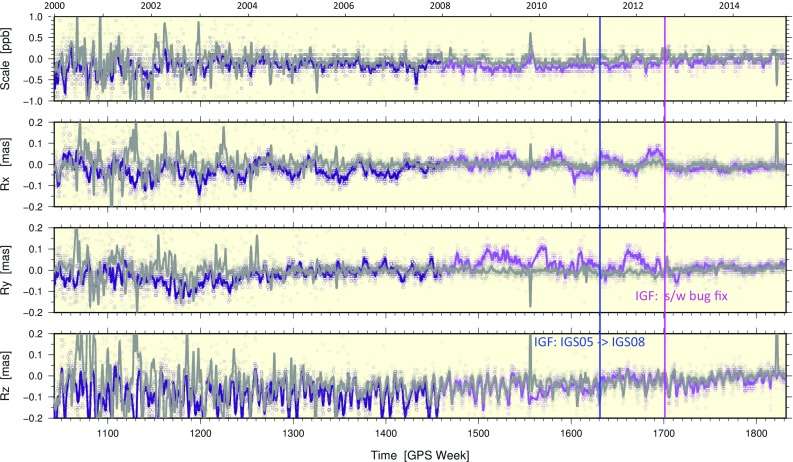
Same as Fig. 10 but for rotation and scale

The time series of PPP-derived Helmert parameters for the daily networks are shown in Figs. 10 and 11 with ensemble statistics summarized in Table 7. Like the position residuals, the frame parameters based on IG2 orbits and clocks are also clearly more scattered than for IG1/F, especially before GPS Week 1336. This is evident in the standard deviations shown in Table 7. In Fig. 10, the convergence of the IG2 and IG1/F Tz series after adopting IGS08 in the IGS operational products is consistent with the Tz offset between IGS05 and IGS08. The elimination of spurious Rx and Ry rotations for IG2 compared to IG1/F when the s/w bug fix was implemented at GPS Week 1702 in the IGF operational series (Fig. 11) is consistent with the orbit combination results (Fig. 6a,b).

**Table 7:** Average and standard deviations of Helmert transformation parameters for the same PPP-based coordinate sets as used for the results in Table 6, and also covering two different time spans: full repro2 and since 2005-08-14. The large IG2 rotations and translations are due to frequently high numbers of station rejections. The stations were rejected during the iterative outlier rejection steps because of large biases in the IG2 satellite clocks, for instance, prior to 2005-08-14. In the more recent years, the IG2 products have smaller long-term frame errors than IG1/F, but the frame less stable at short time intervals as indicated by the larger standard deviations in the IG2 frame parameters since 2005-08-14. The instability arises also because of poorer IG2 clocks compared to IG1/F clocks

Product	Scale (ppb)	Rx (${\upmu }$as)	Ry (${\upmu }$as)	Rz (${\upmu }$as)	Tx (mm)	Ty (mm)	Tz (mm)
Full IG2 time span							
IG1/F	$-$ 0.15	$-$ 15.6	$-$ 1.3	$-$ 66.4	0.91	0.23	4.51
	$\pm \,0.21$	$\pm \,42.5$	$\pm \, 49.1$	$\pm \, 72.9$	$\pm \, 1.36$	$\pm \, 1.40$	$\pm \, 2.54$
IG2	$-$ 0.04	0.6	$-$ 1.4	$-$ 35.5	0.25	0.52	0.10
	± 0.63	± 114.8	± 94.0	± 195.1	± 4.77	± 4.48	± 3.37
Since GPS Wk 1336 (2005-08-14)							
IG1/F	$-$ 0.13	$-$ 8.3	18.1	$-$ 52.1	0.78	0.36	3.75
	$\pm \, 0.14$	$\pm \, 35.1$	$\pm \, 37.1$	$\pm \, 55.4$	$\pm \, 0.99$	$\pm \, 1.22$	$\pm \, 2.49$
IG2	$-$ 0.01	$-$ 0.7	$-$ 1.8	$-$ 33.4	0.42	0.14	$-$ 0.06
	$\pm \, 0.27$	$\pm \, 76.1$	$\pm \, 42.0$	$\pm \, 129.6$	$\pm \, 1.57$	$\pm \, 1.83$	$\pm \, 1.65$

## Summary

IG2 GPS orbits and clocks have been derived from the contributions of 9 analysis groups (6 included with nonzero weights) that reprocessed more than 21 years of ground tracking data. All IG2 orbit and clock product files (*ig2wwwwd.sp3*, *ig2wwwwd.clk*), along with the weekly summary reports (*ig2wwww7.sum*), have been adopted by the IGS and are publicly available. These are in addition to the IG2 terrestrial reference frame products, which formed the IGS contribution to ITRF2014 (Rebischung et al. 2016).

The overall inter-AC orbit agreement is at about the $\sim 4\,\hbox {mm}$ WRMS level (1D) with outlier ACs at exceedingly large levels, reaching several centimeters (GRG) to decimeters (ULR). The agreement between IG2 and IG1/F persists at the $\sim 5\,\hbox {mm}$ WRMS level for most of the repro2 time span, until the most recent years when operational AC products were used to extend their submissions uniformly through GPS Week 1831 (February 14, 2015). For those later weeks, the IG2-IG1/F WRMS disagreement drops to 2.5 mm. Missing from this mix, however, is a reprocessed contribution from NGS (as well as SIO), and that is probably the biggest source for the background 2.5-mm WRMS difference between IG2 and IG1/F orbits.

Large rotational offsets of the orbital frame prior to GPS Week 1702 have been eliminated due to a software bug that was fixed in the operational Finals products and inherited by the repro2 combinations. Absence of Earth albedo and satellite antenna thrust models by ESA dampens an expected 0.72 ppb scale change due to these effects. The scale shift realized in the IG2 orbits was $\sim 0.55$ ppb, leaving a $\sim 0.17$ ppb residual error when IG2 orbits are used and Earth albedo and antenna thrust models are adopted by the user.

A long-arc analysis was performed as a measure of IG2 orbit precision. The root square difference of long-arc post-fit residuals for the IG2 and IG1/F orbits indicates an increased uncertainty of $\sim 7.3\,\hbox {mm}$ (1D RMS) for IG2. However, while less precise than IG1/F, the IG2 orbits offer at least two advantages in a long-term reprocessing: they are internally more self-consistent over the full history, and the orbital frame is much better aligned to the ITRF. The $\sim 0.17$ ppb orbit scale error present should be mitigated in applications using network processing with either double differencing or explicit clock estimation. The same is probably true for mitigating the effects of IG2 lower precision orbits.

The satellite clocks are severely limited by large residual biases and incompatible satellite attitude models adopted by the ACs, and therefore the IG2 clocks should not be used for long-term PPP reprocessing.

Recommendations for the next IGS reprocessing are based on results from this study, along with results from other published work as cited below for the sake of completeness, and include:

- Need improved orbit modeling and AC participation
- Forego clock submission/combination if number and quality of submissions are insufficient for robust combined product, including 30-s satellite clocks ideally
- Need improved models for subdaily variations in Earth orientation due to ocean tides (Griffiths and Ray 2013)
- Full implementation of IERS Conventions by all ACs (with changes below)
- Modify IERS mean pole model to older linear form to agree with Wahr et al. (2015)
- Adopt model for seasonal variations of the low-degree geopotential terms, preferably in agreement with International Laser Ranging Service (ILRS) and International Doris Service (IDS) (e.g., Melachroinos et al. 2014)
- Enforce a common improved satellite attitude model for all ACs to improve inter-AC clock consistency, especially during satellite Earth eclipses
- Require full consistency with IGS and updated IERS Conventions by all ACs
- Establish well-defined analysis standards and reject non-compliant AC solutions

As discussed in the introduction of this paper, other improvements at GNSS tracking stations could benefit IGS products overall, for example by mitigating multipath effects (especially from near-field reflectors), reducing unnecessary discontinuities due to equipment changes and enabling thermal expansion corrections to be computed by collecting the relevant metadata, including but not limited to monument dimensions, materials properties, descriptions of cabling lengths above and below ground.

## Footnotes

1 A test was performed to quantify the impact of including/excluding MIT orbits from the combination. Including MIT orbits after GPS Wk 1249 did not degrade the combination with respect to the IG1/IGF. Apparently, there are sufficient AC inputs to largely suppress the ocean tidal geopotential errors contributed by the MIT orbits, but not the large orbit scale issues prior to GPS Wk 1249. The issues around including/excluding MIT were discussed with the IGS Analysis Center Coordinator priori to finalizing the IG2 combinations; no concerns were raised with the proposal to include MIT after GPS Week 1249.

2 The sensitivity decreases in proportion to the square root of the range. For GPS SVN35, a 1-mm SLR error is consistent with an orthogonal position offset of 199.5 m to 202.3 m, depending on its position in the slightly elliptical orbit.
